# Molecular cloning and expression of bovine nucleoplasmin 2 (NPM2): a maternal effect gene regulated by miR-181a

**DOI:** 10.1186/1477-7827-9-40

**Published:** 2011-03-29

**Authors:** Brandon M Lingenfelter, Swamy K Tripurani, Jyothsna Tejomurtula, George W Smith, Jianbo Yao

**Affiliations:** 1 Laboratory of Animal Biotechnology and Genomics, Division of Animal and Nutritional Sciences, West Virginia University, Morgantown, WV 26506, USA; 2 Laboratory of Mammalian Reproductive Biology and Genomics, Michigan State University, East Lansing, Michigan 48824, USA; 3 Department of Animal Science, Michigan State University, East Lansing, Michigan 48824, USA; 4 Department of Physiology, Michigan State University, East Lansing, Michigan 48824, USA; 5 West Virginia School of Osteopathic Medicine, Lewisburg, WV 24901, USA

## Abstract

**Background:**

Nucleoplasmin 2 (NPM2) is an oocyte-specific nuclear protein essential for nuclear and nucleolar organization and early embryonic development. The aims of this study were to clone the bovine *NPM2 *gene, determine its temporal expression during oocyte development and early embryogenesis, and evaluate the potential role of miRNA-181a in regulation of its expression.

**Methods:**

A 329 bp cDNA fragment was amplified from bovine fetal ovary using primers designed based on the conserved regions of the human and mouse *NPM2 *cDNA sequences. RACE experiments were performed to obtain the 5' and 3' ends of the bovine *NPM2 *cDNA. Real time PCR and Western blot analysis were used to examine the expression of bovine *NPM2 *in oocytes and early embryos. Co-expression of bovine NPM2 and miRNA-181a in Hela cells was performed to determine if expression of bovine NPM2 is regulated by miRNA-181a.

**Results:**

The bovine *NPM2 *cDNA is 851 bp in length encoding a protein of 200 amino acids. The protein contains the conserved bipartite nuclear localization sequence and shows 53% and 62% identity with mouse and human NPM2, respectively. Expression of bovine *NPM2 *mRNA is restricted to ovaries. *NPM2 *mRNA is abundant in GV and MII stage oocytes, decreases in early cleavage stage embryos, and barely detectable in morula and blastocyst stage embryos. Similarly, expression of NPM2 protein is high in oocytes and early embryos but extremely low in blastocysts. The abundance of *NPM2 *mRNA is significantly lower in oocytes isolated from persistent versus growing dominant follicles (*P *< 0.05). A miR-181a binding site in the 3'UTR of the *NPM2 *transcript was identified. Transfection experiments showed that bovine NPM2 protein expression is reduced in Hela cells expressing miR-181a compared to control cells without miR-181a, indicating that translation of NPM2 is repressed by miR-181a.

**Conclusions:**

Our data suggest that expression of bovine NPM2 is temporally regulated during early embryogenesis and miR-181a may play a role in its regulation.

## Background

Maternal mRNAs that accumulate in the oocyte during oogenesis play important roles during initial stages of embryonic development, before activation of the embryonic genome [1]. Some of the maternal transcripts are oocyte-specific and known as maternal effect genes which are required for the early cleavage events post fertilization [2,3]. Examples of maternal effect genes that have been identified in mice include maternal antigen that embryos require (Mater) [4], zygote arrest 1 (Zar1) [5] and nucleoplasmin 2 (Npm2) [6].

To ensure formation of a diploid genome after fertilization, maternal and paternal DNA must undergo remodeling. NPM2, an oocyte-specific nuclear factor, plays an important role in this process. In *Xenopus laevis*, nucleoplasmin (NPM) decondenses sperm DNA after its entry into the oocyte [7,8]. Knockout of NPM2 in mice reduced initial cleavage of embryos and impaired development to the 2-cell stage, and resulted in fragmentation and asynchrony of further cleavage and death by 50 hr post-fertilization [6]. Microinjection of NPM into bovine oocytes after nuclear transfer resulted in increased viability of embryos and higher rate of pregnancy [9], suggesting a role for NPM in facilitating reprogramming of the somatic nucleus.

Degradation of maternal transcripts allows normal embryonic development [10,11]. Multiple mechanisms for maternal RNA degradation exist [12] including the actions of microRNAs (miRNAs). MicroRNAs down-regulate gene expression by binding to known miRNA-target sites on mRNA in the 3' untranslated region (3'UTR) [13]. Knockout of Dicer, an enzyme required for the production of mature miRNAs, results in increased embryonic death in mice [14,15] and abnormal development in zebrafish [16]. A particular miRNA, miR-430, has been showed to target several hundred maternal mRNAs in zebrafish [17].

In domestic animals, major activation of the embryonic genome takes place later as compared to rodents (e.g. 8-16-cell stage in cattle vs. 2-cell stage in mouse) suggesting potential species differences in mechanisms and mediators of the maternal-to-embryonic transition. To date, bovine orthologues of mouse *Mater *and *Zar1 *have been cloned and their expression profiles during oocyte maturation and early embryogenesis characterized [18-20]. Recently, two novel oocyte-specific genes, *JY-1 *and *KPNA7*, have been discovered in cattle and their roles in regulating early embryonic development demonstrated [21,22]. Furthermore, the mechanisms responsible for characteristic temporal expression pattern of products of specific maternal effect genes during early embryogenesis are not completely understood. In this study, we report the cloning of bovine *NPM2*, its mRNA and protein expression during oocyte maturation and early embryonic development and the potential role of miR-181a in regulation of its expression.

## Methods

### Tissue collection and RNA isolation

Bovine tissue samples including adult liver, lung, thymus, kidney, muscle, heart, spleen, cortex (brain), pituitary, adrenal, testis, ovary, and fetal testis and ovaries, were collected at a local slaughterhouse. All samples were frozen in liquid nitrogen and stored at -80°C until RNA isolation. Total RNA was isolated from these tissues using TRIzol reagent (Invitrogen, Carlsbad, CA) and treated with DNase (Promega, Madison, WI) according to manufacturer's protocols.

### RT-PCR analysis of bovine NPM2 mRNA expression

Total RNA from various bovine tissues was used to generate cDNA using oligo (dT)_18 _primer and Superscript III reverse transcriptase (Invitrogen, Carlsbad, CA). Negative control reactions (without the enzyme) were carried out to confirm the absence of genomic DNA contamination. First-strand cDNA was used as a template for PCR amplification of a 329 bp fragment using bovine *NPM2 *gene-specific primers (Additional file 1, Table S1). The PCR was performed using 35 cycles of 94°C for 30 sec, 59°C for 45 sec and 72°C for 30 sec, and a final extension at 72°C for 10 min. Bovine ribosomal protein L19 (*RPL19*) gene was used as a positive control.

### Cloning of bovine NPM2 cDNA by PCR and RACE

PCR primers were designed based on conserved regions of human (NM_182795) and mouse (NM_181345) *NPM2 *sequences to amplify a 329 bp cDNA fragment from a fetal ovary sample (230 days of gestation). The product was cloned using TOPO^® ^TA cloning kit (Invitrogen, Carlsbad, CA) and sequenced. Primers for 5' and 3'RACE were designed based on the obtained bovine *NPM2 *cDNA sequence (Additional file 1, Table S1). RACE experiments were performed to obtain the 5' and 3' ends of bovine *NPM2 *cDNA using the second generation 5'/3'RACE kit (Roche Diagnostics, Indianapolis, IN) following the manufacturer's protocol. Total RNA from bovine fetal ovary was used to generate cDNA with either a gene-specific primer (5'RACE) or an oligo d(T)-anchor primer (3'RACE) followed by nested PCR using gene-specific primers in conjunction with d(T) anchored primers provided in the kit. The specific PCR products were cloned using the TOPO^® ^TA cloning kit (Invitrogen, Carlsbad, CA). The RACE products were sequenced at the University of Illinois Core DNA Sequencing Facility (Urbana, IL).

### Quantitative real time PCR analysis

Expression of bovine *NPM2 *mRNA during oocyte maturation and early embryonic development was determined by real time PCR as described previously [23] using primers listed in Additional file 1, Table S1. GV and MII stage oocytes, pronuclear, 2-cell, 4-cell, 8-cell, 16-cell, morula and blastocyst stage embryos (n = 5 pools of 10 each) were obtained by in vitro fertilization of abattoir derived oocytes as described [23]. Total RNA from oocytes and embryos was isolated using the RNAqueous^®^-Micro kit (Ambion Inc., Austin, TX). Spiked green fluorescent protein (*GFP*) synthetic RNA was used as an exogenous control to account for variations in RNA recovery and efficiency of cDNA synthesis between samples.

Quantitative real time PCR analysis of bovine *NPM2 *mRNA expression in oocytes from growing and persistent dominant follicles was performed as described previously [24]. Oocytes from growing (day 6) and persistent follicles (day 13, estrus = day 0) were used in this analysis. Total RNA isolated from individual oocytes was subjected to linear amplification before real time PCR assay. Bovine *HIST2H2AA4 *gene (BF076713) was used as an endogenous control for data normalization as expression of this gene does not differ in oocytes from the two types of follicles [24]. Primers for this gene are listed in Additional file 1, Table S1.

Quantitative real time PCR analysis of miR-181a expression in oocytes and early stage embryos was performed as described [25]. miR-125b was used as an endogenous control to normalize the target miRNA because this miRNA is expressed consistently in preimplantation mouse embryos [26].

### Western blot analysis

Western blot analysis of bovine NPM2 protein expression in oocytes and early embryos was performed as previously described [22]. The oocyte and embryo samples (50 oocytes/embryos each lane) were purchased from Bomed Inc. (Madison, WI). The primary antibody (anti-bovine NPM2) was prepared commercially by GenScript Corporation (Piscataway, NJ). It was generated by immunizing rabbits with a 15-amino acid synthetic peptide (ERPTWTFKPQKVGKC, amino acid position 26-39) of bovine NPM2 protein. Unpurified antiserum from the third bleed was used in the study.

### Preparation of expression constructs

The plasmid expressing bovine NPM2 was constructed by cloning of the full length bovine *NPM2 *cDNA into pcDNA3.1 expression vector (Invitrogen, Carlsbad, CA). PCR primers (Additional file 1, Table S1) containing BamHI (forward) and XhoI (reverse) restriction sites were designed to amplify the full length bovine *NPM2 *cDNA using a fetal ovary cDNA sample (230 days of gestation). The amplified PCR product was cloned using TOPO^® ^TA cloning kit (Invitrogen, Carlsbad, CA) followed by subcloning into the BamHI and XhoI sites of pcDNA3.1. The plasmid designed to express the bovine miR-181a was prepared by cloning a 262 bp genomic fragment containing the pre-miR-181a into pcDNA3.1. Primers containing BamHI (forward) and PmeI (reverse) restriction sites (Additional file 1, Table S1) were used to amplify the 262 bp DNA fragment from kidney genomic DNA. Following TA cloning the product was cloned into the BamHI and PmeI sites of pcDNA3.1. Both constructs were sequenced to ensure that no mutations were introduced during PCR amplification.

### Cell culture and transfection experiments

HeLa cells from ATCC (Manassas, VA) were grown at 37°C in a humidified incubator containing 5% CO_2 _in Dulbecco's modified Eagle's medium (DMEM; Invitrogen, Carlsbad, CA) supplemented with 10% fetal bovine serum (FBS) and 1% penicillin-streptomycin. Stable HeLa cells expressing bovine miR-181a was generated by transfecting the cells with miRNA-181a plasmid followed by selection in G418. Transfection experiments (n = 5) were conducted using 6-well culture dishes. HeLa cells were passed at least 12 hours prior to all transfection experiments. HeLa cells expressing bovine miR-181a were transfected with *NPM2 *plasmid (1 μg) or pcDNA3.1 vector (1 μg) using FuGene^® ^6 transfection reagent (Roche Applied Science, Indianapolis, IN) according to the manufacturer's instructions. Control Hela cells not expressing bovine miR181a were also transfected with NPM2 plasmid or pcDNA3.1 vector. Twenty-four hours post-transfection, all cells were harvested by trypsinization. Cell pellets were lysed in cell lysis buffer (100 mM sodium β-glycerophosphate, 20 mM HEPES, 15 mM MgCl_2_, 5 mM EGTA, 100 mM 4-amidinophenylmethylsulfonyl fluoride, 3 mg/ml leupeptin, and 10 mg/ml aprotinin, pH 7.5) followed by sonication using an Ultrasonic Homogenizer (BioLogics, Manassas, VA). Western blot analysis of NPM2 protein expression in these cells was performed using anti-bovine NPM2 antibody as described above. Detection of GAPDH (protein loading control) was performed using anti-GAPDH antibody (Ambion, Austin, TX). Density of the protein bands was determined using a densitometer and NPM2 protein expression was normalized with GAPDH.

### Statistics

Differences in expression of *NPM2 *mRNA and protein and miRNA-181a between samples were analyzed by GLM procedures of SAS with LS means (SAS 9.1.3, SAS Institute Inc., Cary, NC). Different letters indicate significant differences (*P *< 0.05).

## Results and discussion

### cDNA cloning and tissue distribution of bovine NPM2

**Figure 1:**
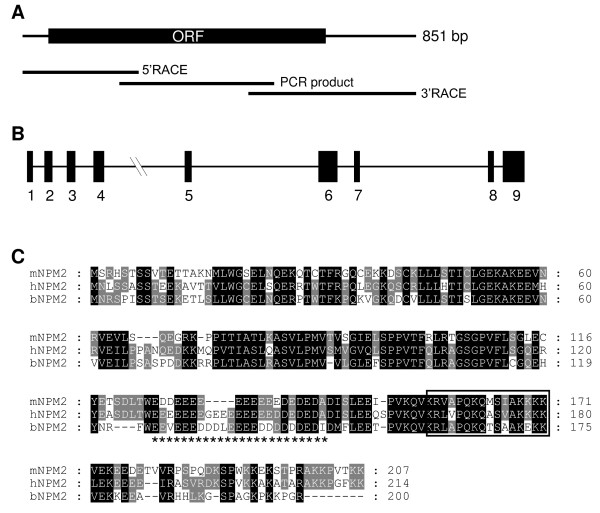
**Cloning and sequence analysis of bovine *NPM2 *gene**. **A**. Schematic representation of bovine *NPM2 *cDNA and its cloning strategy. **B**. Schematic representation of bovine *NPM2 *gene structure. **C**. Multiple alignment of the deduced amino acid sequence of bovine NPM2 with its human and mouse counterparts. Alignment of amino acid sequences from human (NP_877724), mouse (NP_851990) and bovine (ACT65735) NPM2 proteins was performed using ClustalW analysis [42]. The area indicated by the box is the nuclear localization signal. The stars in the alignment represent the putative histone binding region [a stretch of negatively charged glutamic acid (E) and aspartic acid (D) residues].

The complete cDNA sequence (851 bp) for bovine *NPM2 *was obtained by assembly of the sequences from the PCR and RACE fragments (Figure 1A). The sequence has been deposited in GenBank (accession number: FJ769182). BLAST search of the bovine reference genome sequence in the NCBI database using the bovine *NPM2 cDNA *did not find the corresponding gene sequence. However, a previous bovine chromosome 8 genomic contig sequence (NW_932049) in GenBank database contains the *NPM2 *gene which spans ~14.6 kb. Alignment of the cDNA with the genomic sequence using the NCBI Splign program [27] revealed that the bovine *NPM2 *gene contains 9 exons separated by 8 introns (Figure 1B). Visual inspection of the promoter region identified 2 putative E-boxes (cacctg) located approximately 200 bp upstream of the predicted transcription start site (data not shown). Such elements are known to be responsible for oocyte-specific gene expression [28,29].

The bovine *NPM2 *cDNA encodes a protein of 200 amino acids. Analysis of the predicted NPM2 protein sequence revealed that it contains a conserved bipartite nuclear localization signal (**KR**LAPQKQTSAA**KEKK**), and a series of glutamic and aspartic residues (E and D) implicated in binding histones and protamines (Figure 1C). The protein shows 53% and 62% amino acid sequence identity with the mouse and human NPM2, respectively (Figure 1C).

**Figure 2:**
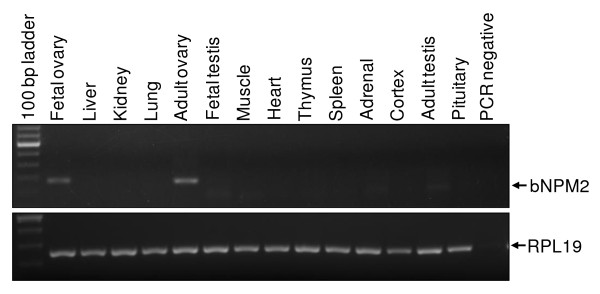
**Tissue distribution of bovine *NPM2 *mRNA**. Expression of *NPM2 *mRNA in various bovine tissues including liver, lung, thymus, kidney, muscle, heart, spleen, cortex, pituitary, adrenal, testis, ovary, fetal testis and fetal ovary was determined by RT-PCR analysis. Bovine ribosomal protein L19 (*RPL19*) was used as a positive control for RNA quality and RT.

To determine the tissue distribution of the bovine *NPM2 *gene, RT-PCR was performed using RNA samples isolated from multiple bovine tissues. As shown in Figure 2, expression of bovine *NPM2 *mRNA was restricted to adult and fetal ovaries, which is consistent with the result of Northern blot analysis to detect mouse *Npm2 *expression [6].

### Expression of bovine NPM2 mRNA and protein in oocytes and early embryos

**Figure 3:**
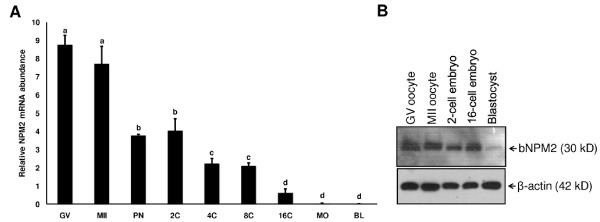
**Expression of bovine *NPM2 *mRNA and protein during oocyte maturation and early embryonic development**. **A. **Real time PCR analysis of bovine *NPM2 *mRNA expression during *in vitro *oocyte maturation and early embryogenesis (mean ± SEM, n = 4 pools of oocytes or embryos). The oocytes and embryo samples used in the experiment included GV- and MII-stage oocytes, pronuclear (PN), 2-cell (2C), 4-cell (4C), 8-cell (8C), 16-cell (16C), morula (MO)-stage, and blastocyst (BL)-stage embryos. Data were normalized relative to abundance of *GFP *RNA (exogenous control). Different letters indicate statistical difference (*P *< 0.05). **B**. Determination of bovine NPM2 protein expression in GV- and MII-stage oocytes, and early embryos at 2-cell, 16-cell and blastocyst stage by Western blot analysis using anti-bovine NPM2 antibodies.

Expression of *NPM2 *mRNA in bovine oocytes (GV and MII stage) and early embryos (pronuclear, 2-cell, 4-cell, 8-cell, 16-cell, morula and blastocyst stage) was investigated by quantitative real time PCR. The results indicate that *NPM2 *mRNA is most abundant in GV and MII stage oocytes relative to early embryos. The abundance of mRNA for NPM2 is decreased in embryos at pronuclear and 2-cell stage, is further decreased from 4-cell to 16-cell stages, reaching a level that is barely detectable in morula and blastocyst stage embryos (Figure 3A; P < 0.05). Abundance of bovine NPM2 protein in GV and MII stage oocytes as well as in 2-cell, 16-cell and blastocyst stage embryos was evaluated by Western blot analysis. As shown in Figure 3B, the protein is abundant in GV and MII stage oocytes and remains fairly abundant in 2-cell and 16-cell stage embryos but drops sharply by the blastocyst stage. The immunoreactive protein band for bovine NPM2 protein is approximately 30 kDa which is similar to the size of mouse NPM2 protein (32 kDa) [30] but larger than predicted size of ~22 kDa. Sumoylation of the protein could be a cause for this discrepancy as the protein contains a sumoylation consensus motif (ΨKXE). The expression pattern of bovine *NPM2 *mRNA and protein during early embryogenesis is very similar to a number of known bovine maternal effect genes essential for early embryonic development [18,19,21,22] and suggests maternal origin of this factor. Furthermore, *NPM2 *mRNA abundance in 8-cell embryos was not diminished by culture in the presence of the transcriptional inhibitor alpha-amanitin (data not shown) indicating that the *NPM2 *mRNA detected in the early bovine embryos is maternal/oocyte derived. Although the *NPM2 *mRNA was significantly reduced by the 16-cell stage, the protein level did not decrease until after the 16-cell stage (Figure 3B), indicating that NPM2 may be required for nuclear reorganization in embryos through the time of embryonic genome activation in cattle at the 8- to 16-cell stage [31].

### Expression of bovine NPM2 mRNA is decreased in oocytes from persistent follicles

**Figure 4:**
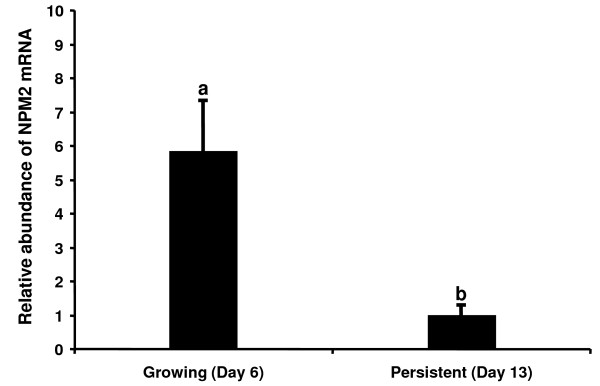
**Determination of *NPM2 *mRNA abundance in oocytes from growing and persistent dominant follicles by quantitative real time PCR analysis (mean ± SEM, n = 4)**. Bovine *HIST2H2AA4 *gene was used as an endogenous control for data normalization. Relative abundance of *NPM2 *mRNA is significantly lower in oocytes from persistent than from growing follicles. Different letters indicate statistical difference (*P *< 0.05).

Results of numerous studies indicate that competence or quality of oocytes harvested from persistent dominant follicles is poor [32,33]. For example, embryos derived from oocytes obtained from persistent dominant follicles underwent embryonic death in vivo by the 16-cell stage [34]. Our previous experiments demonstrated that mRNA abundance for genes (*MSY2*, *PARN *and *YY1*) important for early embryogenesis was significantly lower in oocytes obtained from persistent versus growing dominant follicles [24]. As NPM2 is a key oocyte-specific nuclear factor essential for early embryonic development, the relative abundance of bovine *NPM2 *mRNA in oocytes from growing (day 6) and persistent follicles (day 13) was determined by real time PCR. The results indicate that the level of bovine *NPM2 *mRNA is significantly lower in oocytes from persistent versus growing dominant follicles (*P *< 0.05) (Figure 4), supporting a potential relationship between *NPM2 *mRNA abundance and oocyte competence. This result is also consistent with results in rainbow trout where low-quality eggs were shown to have lower levels of *NPM2 *transcript [35]. It is likely that insufficient oocyte store of *NPM2 *mRNA results in decreased ability of oocyte to perform sperm chromatin remodeling at fertilization which is essential for early embryogenesis. As the expression of NPM2 is associated with oocyte quality, it could potentially serve as a marker for oocyte developmental competence. The relationship between oocyte quality and *NPM2 *mRNA abundance merits further investigation in other models of oocyte quality.

### Evidence of translational silencing of bovine NPM2 by miR-181a

**Figure 5:**
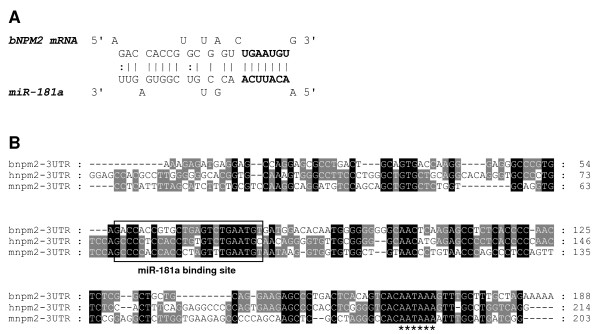
**Prediction of a miR-181a binding site in the 3'-UTR of *NPM2 *cDNA**. **A**. Predicted miR-181a binding site in bovine *NPM2 *3'-UTR. **B**. Alignment of the 3'-UTR sequences from human, mouse and bovine *NPM2 *cDNA sequences (alignment was performed using ClustalW analysis). The outlined box indicates the conserved miR-181a binding site.

Based on the expression pattern of bovine *NPM2 *mRNA during early embryonic development and recent evidence indicating the involvement of miRNAs in regulation of maternal RNA [15-17], it was hypothesized that NPM2 might be targeted by miRNAs for silencing and/or degradation during early embryogenesis. Potential miRNA binding sites in the 3'UTR of bovine *NPM2 *mRNA were predicted using MicroInspector [36], an algorithm for detection of possible interactions between miRNAs and target mRNA sequences [37]. A miR-181a binding site in bovine *NPM2 *3'UTR was identified (Figure 5A). Alignment of the 3'UTR sequence for bovine *NPM2 *mRNA with the human and mouse orthologous sequences revealed that the "seed" sequence of the binding site, corresponding to the most important region for miRNA:mRNA interactions, was conserved in all aligned sequences (Figure 5B).

**Figure 6:**
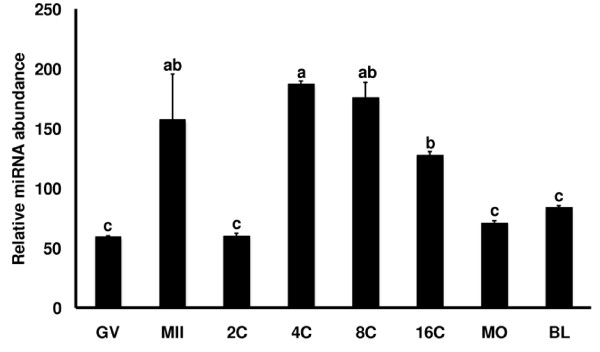
**Quantitative real-time PCR analysis of miR-181a expression in oocytes and early embryos (mean ± SEM, n = 3)**. The oocytes and embryo samples in the experiment included GV- and MII-stage oocytes, 2-cell (2C), 4-cell (4C), 8-cell (8C), 16-cell (16C), morula (MO)-stage, and blastocyst (BL)-stage embryos. Quantity of miR-181a was normalized to miR-125b. Different letters indicate statistical difference (*P *< 0.05).

An initial experiment was conducted using real time PCR to determine if miR-181a is expressed in oocytes and early embryos. As shown in Figure 6, miR-181a is present in all stages of oocytes and early embryos examined. There appears to be an increase in miR-181 in oocytes during the transition from GV to MII stage. Following a drop in 2-cell stage embryos, the level of miR-181a increases again in embryos at 4-cell to 16 cell stage, a period corresponding to the time of embryonic genome activation in cattle when expression of NPM2 decreases gradually. The inverse correlation between miR-181a and NPM2 expression during early embryogenesis further supports our hypothesis that NPM2 might be down-regulated by miR-181a.

**Figure 7:**
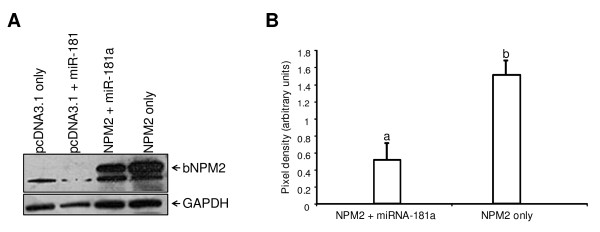
**Western blot analysis of NPM2 protein expression in Hela cells transfected with *NPM2 *and miR-181a expression plasmids**. **A**. A representative Western blot showing suppression of NPM2 protein in cells expressing miR-181a. **B**. Densitometric analysis of protein bands on the Western blots. Data were normalized to GAPDH and expressed as relative pixel density (mean ± SEM, n = 5). Different letters indicates statistical significance (*P *< 0.05).

To test if miR-181a regulates NPM2 protein expression in the context of its native mRNA sequence, co-expression studies using a construct containing the full length bovine *NPM2 *cDNA and a plasmid designed to deliver bovine miR-181a were performed. Western blot analysis using antibodies against bovine NPM2 showed that expression of bovine NPM2 protein was reduced in cells expressing miR-181a compared to control cells without miR-181a (Figure 7A, the minor band present is all samples apparently is a non-specific signal). Quantification of NPM2 protein expression using densitometry (Figure 7B) confirmed that co-expression with miR-181a decreased the level of NPM2 protein (*P *< 0.05), indicating that translation of NPM2 is repressed by miR-181a.

miR-181a is a conserved miRNA that has been identified in diverse species. Recent studies have suggested that miR-181a may function as a tumor suppressor in cancer cells [38] or a modulator of cisplatin-induced cancer cell death [39]. It has also been reported that miR-181a down regulates the expression of zinc finger (ZNF) genes by targeting the sequences coding for the ZNF C2H2 domain [40]. The involvement of miR-181a in regulating the expression of NPM2 supports a new role of this miRNA in early embryonic development. Specific miRNAs known to be responsible for maternal mRNA degradation have been reported in zebrafish [17] and Xenopus [41]. However, in mammals, specific miRNAs targeting maternal effect genes have not been reported. This study represents the first report of a specific miRNA potentially involved in regulation of a maternal effect gene in mammalian species.

## Conclusions

Our data suggest that bovine NPM2 present in early embryos is of maternal origin and NPM2 is positively associated with oocyte developmental competence. Results also demonstrate suppression of NPM2 translation by miR-181a and suggest that expression of this essential nuclear factor during early embryogenesis is potentially regulated by miR-181a.

## Competing interests

The authors declare that they have no competing interests.

## Authors' contributions

BML, SKT and JT performed the experiments. BML drafted the manuscript. GWS and JY designed the study, supervised the experimental work and revised the manuscript. All authors read and approved the final manuscript.

## Supplementary Material

Additional file 1 **Table S1: List of primers used in the study**. Click here for file
